# Macaques as model hosts for studies of HIV-1 infection

**DOI:** 10.3389/fmicb.2013.00176

**Published:** 2013-06-28

**Authors:** Anisha Misra, Rajesh Thippeshappa, Jason T. Kimata

**Affiliations:** Department of Molecular Virology and Microbiology, Baylor College of MedicineHouston, TX, USA

**Keywords:** HIV-1, SIV, AIDS, macaque models, tropism, innate restriction

## Abstract

Increasing evidence indicates that the host range of primate lentiviruses is in part determined by their ability to counteract innate restriction factors that are effectors of the type 1 interferon (IFN-1) response. For human immunodeficiency virus type 1 (HIV-1), *in vitro* experiments have shown that its tropism may be narrow and limited to humans and chimpanzees because its replication in other non-human primate species is hindered by factors such as TRIM5α (tripartite motif 5 alpha), APOBEC3G (apolipoprotein B mRNA-editing, enzyme-catalytic, polypeptide-like 3), and tetherin. Based on these data, it has been hypothesized that primate lentiviruses will infect and replicate in a new species if they are able to counteract and evade suppression by the IFN-1 response. Several studies have tested whether engineering HIV-1 recombinants with minimal amounts of simian immunodeficiency virus sequences would enable replication in CD4^+^ T cells of non-natural hosts such as Asian macaques and proposed that infection of these macaque species could be used to study transmission and pathogenesis. Indeed, infection of macaques with these viruses revealed that Vif-mediated counteraction of APOBEC3G function is central to cross-species tropism but that other IFN-induced factors may also play important roles in controlling replication. Further studies of these macaque models of infection with HIV-1 derivatives could provide valuable insights into the interaction of lentiviruses and the innate immune response and how lentiviruses adapt and cause disease.

## INTRODUCTION

Early studies on primate lentiviruses identified key host cell factors required for replication (Hatziioannou and Evans, 2012). More recent investigations have shown that overcoming the suppressive effects of innate restriction factors is also necessary for human immunodeficiency virus type 1 (HIV-1) and simian immunodeficiency viruses (SIVs) to replicate in human and non-human primate hosts, respectively. Viral accessory proteins play key roles in antagonizing these inhibitory factors, which are effectors of the type 1 interferon (IFN-1) response (Harris et al., 2012). However, their functional activities are commonly limited to susceptible host species, suggesting that innate immunity may be a significant barrier to transmission of lentiviruses. We, and others, have engineered HIV-1 recombinants with minimal SIV sequences conferring resistance to specific restriction factors and infected macaques to experimentally test this hypothesis. Investigations utilizing these macaque-tropic HIV-1 derivatives may lead to a greater understanding of inter-species transmission of primate lentiviruses as well as the development of a macaque model of HIV-1 infection and disease.

## MACAQUE AIDS MODEL DEVELOPMENT AND SPECIES TROPISM OF PRIMATE LENTIVIRUSES

The development of non-human primate acquired immunodeficiency syndrome (AIDS) models provided initial insights into the species tropism of lentiviruses. In particular, these experiments demonstrated a narrow species tropism for HIV-1. Gibbons and chimpanzees are susceptible to HIV-1 (Gardner and Luciw, 1989; Fultz, 1993). However, due to their endangered status and maintenance cost, they are not reasonable model hosts. On the other hand, Asian macaques, including *Macaca mulatta* (rhesus macaques, RM) and *M. fascicularis* (cynomolgus monkeys, CM) and cells from these species appear to be resistant to HIV-1 (Agy et al., 1992; Cowan et al., 2002; Munk et al., 2002), suggesting genetic barriers to infection. In retrospect, these findings are not surprising given that HIV-1 evolved from a novel recombinant SIV infecting chimpanzees (SIVcpz; Gao et al., 1999; Bailes et al., 2003). Uniquely, one species, *M. nemestrina* (pigtailed macaques, PTM), has been found to be susceptible to transient infection but not disease (Agy et al., 1992, 1997; Gartner et al., 1994), demonstrating that a potent resistance mechanism(s) may indeed control viral replication.

With the absence of a susceptible non-human primate host for HIV-1, a SIV-AIDS macaque model was developed accidently following the discovery that Asian macaques housed with sooty mangabeys at a US primate center had developed AIDS like disease (Gardner, 1996; Apetrei et al., 2005). Although African monkey species harbor SIVs and live with high virus loads without developing disease (Klatt et al., 2012b), SIVs isolated from sooty mangabeys (SM, *Cercocebus atys*) cause AIDS at varying rates when inoculated into Asian macaques. As a result, SIV infection of macaques has become the most widely used model for studies of AIDS immunopathogenesis and viral fitness (Kimata, 2006; Hatziioannou and Evans, 2012). Quite interestingly, PTMs appear to be more susceptible to infection and disease induced by SIV than RMs, which may be due to a higher level of immune activation and gastrointestinal immune dysfunction (Klatt et al., 2012a; Canary et al., 2013).

**FIGURE 1:**
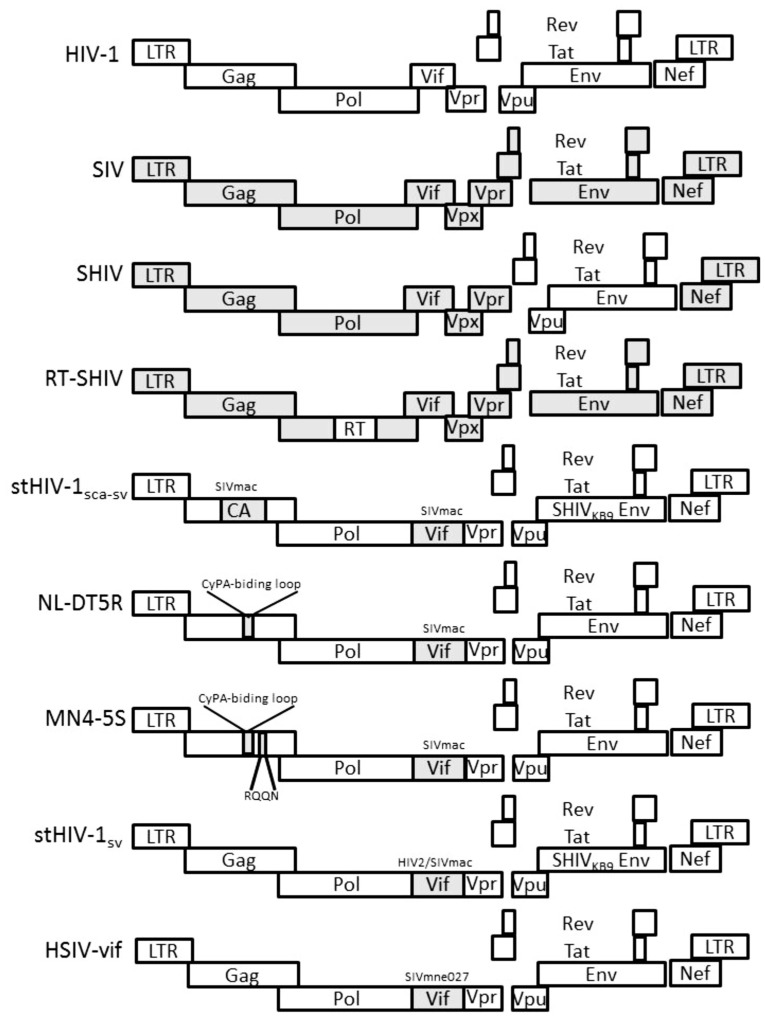
**Genetic organization of HIV-1, SIV, and HIV-1/SIV chimeric proviruses.** HIV-1 sequences are in white. SIV sequences are shaded gray.

Genetic differences in reverse transcriptase and protease of HIV-1 and SIVmac make it difficult to evaluate the efficacy of antiretroviral drugs that target these proteins using the SIVmac-RM model. Evaluating vaccines against HIV-1 is also impossible since cytotoxic T cell epitopes may differ and neutralizing antibodies are not cross-reactive. These shortcomings have been partially addressed by constructing chimeric SIV/HIV-1 viruses (SHIVs) that include certain HIV genes in the SIVmac239 backbone (Shibata et al., 1991; **Figure 1**).

Aside from the obvious utility for translational studies, the development of SHIVs revealed important clues about the functional activity of HIV-1 proteins in macaques. SIVmac based chimeras that include HIV-1 gene substitutions in *env*, *tat*, and *rev* (Env-SHIV) or *nef* (Nef-SHIV) are pathogenic in macaques (Li et al., 1995; Luciw et al., 1995; Reimann et al., 1996; Sinclair et al., 1997; Alexander et al., 1999). Chimeras with HIV-1 *rt* substitutions (RT-SHIVs) also persistently replicate in macaque hosts (Uberla et al., 1995; Ambrose et al., 2007). While not required, *vpu *of HIV-1 enhances the pathogenicity of Env-SHIV (Stephens et al., 2002). Thus, a significant amount of HIV-1 sequences can functionally replace SIV sequences, but determinants within *gag*–*pol* and *vif* of SIV appear necessary for infection of Asian macaques.

## INNATE RESTRICTION FACTORS OF PRIMATE LENTIVIRUSES

Several cellular restriction factors have been identified that can limit replication of primate lentiviruses in different species, but whose activities are specifically inhibited or evaded (**Table 1**). These include apolipoprotein B mRNA-editing, enzyme-catalytic, polypeptide-like 3 (APOBEC3) proteins, tripartite motif 5 alpha (TRIM5α) and related TRIM5–cyclophilin A fusion proteins (TRIMcyp), tetherin/BST2/CD317, and sterile alpha motif (SAM) domain and HD domain-containing protein 1 (SAMHD1; Thippeshappa et al., 2012). All are regulated by IFN-1, suggesting that innate immunity plays a critical role in preventing infection and that viral adaptations that antagonize or escape the effects of the factors may be required for successful transmission of lentiviruses.

**Table 1:** Restriction factors and primate lentivirus infection.

Restriction factors	Mechanism of inhibition	Inhibitory activity in different species
TRIM5α	Binds viral capsid and blocks infection at or before reverse transcription; innate immune sensing of retroviral infection	TRIM5α blocks HIV-1 infection of Asian macaques, except PTM, which do not express TRIM5α; Allelic variation in TRIM5α influences control of SIVs in RM; viral capsid mutations confer resistance to TRIM5α
TRIMcyp		RM and PTM TRIMcyp do not inhibit HIV-1; variation in CMTRIMcyp influences inhibition of HIV-1, HIV-2, and SIVagm, but not SIVmac; viral capsid mutations enable evasion in susceptible hosts
APOBEC3 family proteins	Introduce G to A mutations, reduce infectivity, interfere with reverse transcription	Blocks HIVs and SIVs in the absence of Vif in non-human primates and humans; Vif inhibitory activity against APOBEC3 proteins is limited to virus-adapted host species
BST2/tetherin	Restricts release of virions from the cell surface; innate sensing of infection and promotion of inflammatory responses	Inhibits virion release from human and non-human primate cells; HIV-1 Vpu, SIV Nef, and HIV-2 Env antagonize tetherin only in virus-adapted species
SAMHD1	Reduces dNTP pool required for cDNA synthesis	SAMHD1 proteins from different non-human primate species and humans inhibit HIV and SIV infection of myeloid derived cells and resting T cells; Vpx and Vpr proteins from some SIVs direct proteasome-mediated degradation of SAMHD1 of virus-adapted non-human primate species and humans; HIV-1 does not antagonize SAMHD1

The APOBEC3 (A3) proteins belong to a seven-member family of cytidine deaminases (Jarmuz et al., 2002). A3G was identified as a Vif-targeted inhibitory factor of HIV-1 during a screen for cellular factors that blocked post-entry steps of infection prior to integration (Sheehy et al., 2002). In the absence of Vif, it interferes with viral replication by incorporating into the virion and disrupting reverse transcription or causing accumulation of deleterious G to A mutations (Mangeat et al., 2003; Zhang et al., 2003; Bishop et al., 2008). Hypermutated viral genomes may be degraded or produce non-functional truncated or misfolded viral proteins that are processed and serve as antigens for cellular immune responses (Casartelli et al., 2010).

In virus producing cells, Vif binds A3G and links it to an E3 ubiquitin ligase complex, thereby redirecting it for degradation by the proteasome (Conticello et al., 2003) and preventing its incorporation into assembling virions. Interestingly, Vif function appears to be species-specific. For example, the HIV-1 Vif antagonizes the human A3G protein but not A3G of other non-human primate species. By contrast, the Vif protein of SIVagm antagonizes African green monkey (AGM) A3G but not human A3G (Mariani et al., 2003). These findings suggest that Vif-mediated inhibition of the A3G proteins is likely essential for transmission of a virus to a new host species.

Of the innate restriction factors, only TRIM5α was initially discovered as an inhibitory factor of HIV-1 in Old World Monkeys (OWMs; Stremlau et al., 2006; Grutter and Luban, 2012). TRIM5α blocks a post-entry stage of HIV-1 replication through an interaction with the capsid protein. It belongs to the tripartite family of proteins, and contains a RING finger, B-box2, and coiled coil domain, which are responsible for E3 ubiquitin ligase activity and higher order self-association. It also has a B30.2/SPRY domain that detects the incoming viral capsid proteins, linking the viral core to an ubiquitin-proteasome-dependent pathway. This disrupts the preintegration complex, thereby blocking reverse transcription. However, in cases where the proteasome pathway is inhibited, nuclear entry of viral DNA is impaired. Recent studies also establish TRIM5α as an innate immune sensor of the retroviral capsid (Pertel et al., 2011). Sequence variation in B30.2/SPRY of TRIM5α and amino acid variations in the viral capsid are responsible for species-specific restriction and evasion, respectively (Nakayama et al., 2005; Sawyer et al., 2005). Additionally, allelic variation in TRIM5 influences transmission and modulates disease progression in SIV-infected RM (Kirmaier et al., 2010; Lim et al., 2010b; Reynolds et al., 2011). Interestingly, PTMs do not express a TRIM5α isoform, partially explaining their unique susceptibility to HIV-1 (Brennan et al., 2007).

Novel TRIMcyp also interfere with post-entry steps in HIV/SIV infection. First identified in New World Owl Monkeys (Sayah et al., 2004), the fusion protein appears to have arisen via line-mediated retrotransposition of the cyclophilin A gene into the TRIM5 locus. Subsequent studies have also identified TRIMcyp fusion proteins in RM, CM, and PTM that evolved independently (Brennan et al., 2008; Newman et al., 2008; Virgen et al., 2008; Wilson et al., 2008; Dietrich et al., 2011). Allelic variation in the cyclophilin A domain of the macaque TRIMcyp proteins affects recognition and inhibition of HIV-1 and 2 and SIVagm but not SIVmac. Interestingly, RMs and CMs are polymorphic for TRIM5α alleles and TRIMcyp, although geographically distinct CM populations show different frequencies of TRIMcyp. PTMs, on the other hand, are homozygous for TRIMcyp, again demonstrating a unique genotype for PTMs in comparison to other Asian macaques (Brennan et al., 2008; Newman et al., 2008; Kuang et al., 2009; Dietrich et al., 2011; Saito et al., 2012).

Tetherin or BST2 is interferon inducible type II membrane protein that interferes with the release of HIV-1 progeny virions from the surface of infected human T cells and also functions as an innate immune sensor of viral infection to promote inflammatory responses (Neil et al., 2008; Van Damme et al., 2008; Galao et al., 2012). Initially, it was discovered that the HIV-1 protein Vpu inhibits tetherin and is required for the efficient release of progeny virions (Neil et al., 2008; Van Damme et al., 2008). Subsequent studies have shown that primate lentiviruses that do not encode Vpu evolved other strategies to antagonize tetherin. For example, HIV-2 and SIV use Env- and Nef-dependent mechanisms to counteract the restrictive effect of tetherin (Jia et al., 2009; Le Tortorec and Neil, 2009), respectively. Additionally, the effects of the viral antagonists are specific for the host species in which they evolved. The HIV-1 Vpu evolved to overcome the activity of human tetherin, but it is ineffective against tetherin from chimpanzees, RM, AGM, and mustached monkeys (Jia et al., 2009; Sauter et al., 2009; Lim et al., 2010a; Yang et al., 2010). Despite the close relatedness of HIV-1 and SIVcpz, Vpu of SIVcpz does not antagonize chimpanzee tetherin. Instead it uses Nef to downregulate chimpanzee tetherin expression like other SIVs, which also exhibits species-specific activity (Jia et al., 2009; Zhang et al., 2009).

SAMHD1 is a restriction factor that inhibits HIV-1 infection of myeloid cells (Hrecka et al., 2011; Laguette et al., 2011). Although its exact biological function is unclear, mutations in SAMHD1 can result in Aicardi Goutieres syndrome whose symptoms mimic that of a viral infection (Rice et al., 2009). Vpx protein from either HIV-2 or the SIVsm lineage inhibit human SAMHD1, resulting in its degradation through the proteasome. It has been noted that Vpx expression or SAMHD1 depletion increases the amount of dNTP’s in macrophages, which suggests that SAMHD1 decreases the dNTP pool required for viral cDNA synthesis (Lahouassa et al., 2012). Structural studies also indicate that SAMHD1 is a dNTP triphosphate triphosphohydrolase (Goldstone et al., 2011). Interestingly, SAMHD1 only restricts infection of HIV-1 in non-dividing cells such as macrophages and resting T cells but not activated proliferating T cells (Baldauf et al., 2012; Descours et al., 2012). New data also indicate that phosphorylation may regulate SAMHD1’s restriction activity (Cribier et al., 2013; White et al., 2013).

Like the other restriction factors, Vpx appears to antagonize SAMHD1 in a species-specific manner since human and gibbon SAMHD1 can be degraded by Vpx proteins from HIV-2rod, SIVmac, and SIVsm but not by Vpx from SIVrcm and SIVmnd2. However, Vpx proteins from different SIV and HIV-2 strains can induce degradation of RM and SMSAMHD1 (Laguette et al., 2012; Lim et al., 2012). Interestingly, some SIVs inhibit SAMHD1 of their natural hosts via Vpr. Thus, targeting SAMHD1 appears critical for replication and persistence of SIVs in OWMs. It is therefore interesting that HIV-1 does not have a mechanism to antagonize SAMHD1 in human cells. One hypothesis is that this may help the virus avoid immune sensing.

Other innate restriction factors such as interferon inducible transmembrane proteins (IFITM), and 2′,3′-cyclic-nucleotide 3′-phosphodiesterase (Lu et al., 2011; Wilson et al., 2012) have been shown to interfere with early and late stages of the viral life cycle, respectively. However, whether these factors have species-specific activity against primate lentiviruses is unknown.

## ENGINEERING MACAQUE-TROPIC HIV-1 DERIVATIVES

The species-specific effects of innate restriction factors and requirement for particular SIV sequences for replication competent SHIV chimeras suggested that engineering macaque-tropic recombinant viruses consisting of mainly HIV-1 sequences may be possible as long as the virus can evade or antagonize key host restriction factors (**Figure 1**). Hatziioannou et al. (2006) generated the initial HIV-1 chimera with minimal SIV sequences that could replicate in RM peripheral blood mononuclear cells (PBMCs; stHIV-1_sca-sv_). The virus included *ca* and *vif* substitutions from SIVmac in order to escape restriction by RM TRIM5α and A3G, respectively. In other studies, a macaque-tropic HIV-1 derivative with the SIV *vif* gene and a short 21 base pair segment corresponding to the HIV-1 cyclophilin A binding loop from SIV was constructed (NL-DT5R; Kamada et al., 2006; Igarashi et al., 2007). The virus showed increased infectivity in both CM and PTM T cells. However, only after passaging in a CM T cell line was the virus able to replicate efficiently in CD8^+^ cell-depleted PBMCs from either PTM or RM. While these HIV-1 derivatives infected PTM, they were rapidly controlled and did not cause disease. Additional studies selected *gag* variants better able to escape restriction by CM TRIMcyp (e.g., MN4-5S), but replication only modestly improved in CMs (Kuroishi et al., 2009; Saito et al., 2011).

Because of the absence of a post-entry block to HIV-1 infection and potential for more rapid AIDS progression, PTMs were hypothesized to be the most susceptible to macaque-tropic HIV-1 derivatives. Indeed, substituting *vif* in HIV-1 with alleles from SIVmne (HSIV-vif) or SIVmac or HIV-2 (stHIV-1) is sufficient for HIV-1 to replicate in PTM CD4^+^ T cells (Hatziioannou et al., 2009; Thippeshappa et al., 2011). Infection of PTMs with mtHIV-1 resulted in acute infection and viremia that was controlled within 25 weeks post-infection. Interestingly, replication of HSIV-vif in PTMs extended for over 90 weeks post-infection, although plasma viral loads were low. Moreover, one animal demonstrated a steep drop in CD4^+^ T cell counts, persistent but low viremia, and opportunistic infections after three years of infection (unpublished observations). It will be important to reisolate variants from this animal and examine the genetic and phenotypic changes that have occurred during infection. Since the different variants of HIV-1 and SIV used in these studies seem to make a difference in persistence and disease, other variants should be considered for future *in vivo* infection experiments.

What accounts for virological control in the PTMs remains unclear. There is suggestion from CD8^+^ cell-depletion studies that cellular immune responses may be limiting replication of the macaque-tropic HIV-1 clones (Hatziioannou et al., 2009). Additionally, the IFN-1 response might restrict viral replication. IFNs are upregulated during HIV-1 and SIV infections (Neil and Bieniasz, 2009; Thippeshappa et al., 2012). Thus, these viruses must be able to overcome the induction of restrictive interferon-stimulated genes (ISGs) in order to replicate to high levels and cause disease. Indeed, new studies demonstrate that the prototype macaque-tropic HIV-1 derivatives are inhibited by IFNα in PTM cells. By contrast, pathogenic SIVmne and SIVmac clones are highly resistant to IFNα-induced inhibition (Bitzegeio et al., 2013; Thippeshappa et al., 2013). Interestingly, suppression of replication of the HIV-1 derivatives by IFNα may not be due to the induction of known restriction factors such as tetherin, TRIM5α, TRIMcyp, A3G, or SAMHD1, indicating that other ISGs may be responsible for potently blocking replication of macaque-tropic HIV-1 in PTMs. Furthermore, IFNα resistance may be acquired by mutations in *env*, enabling escape from an early block in replication (Thippeshappa et al., 2013). Infection of PTMs with this variant could provide insight into whether evasion of IFNα is critical for viral replication in the host.

## SUMMARY AND CONCLUSIONS

The engineering of macaque-tropic HIV-1 derivatives has shed light on the significance of counteracting or escaping restriction factors of the innate immune response for cross-species transmission. Macaque models have provided experimental *in vivo* systems to demonstrate the importance of Vif-mediated antagonism of A3 proteins and evasion of TRIM5 isoforms. Indeed, in the absence of inhibitory TRIM5α or TRIMcyp alleles in the PTM, Vif-mediated inhibition of A3G is necessary and sufficient for transmission and persistence of HIV-1 in PTMs. However, the SIV Vif is not sufficient for robust replication of macaque-tropic HIV-1 chimeras in PTMs because these viruses fail to adequately overcome the IFNα-induced antiviral state. Additional adaptations like those we have identified in an *env* sequence may be necessary for HIV-1 to replicate to high levels in the PTM or other macaque hosts. What other restriction factors might play a role in controlling HIV-1 replication in OWMs like Asian Macaques is unclear, but the IFNα resistance mutations may help identify new mechanisms of escape. Finally, it is curious that lentiviruses of OWMs target SAMHD1 for degradation via Vpx or Vpr, and that Vpx enhances transmission and pathogenesis of SIV in PTMs (Hirsch et al., 1998; Belshan et al., 2012), but HIV-1 did not evolve a mechanism to inhibit this protein in humans. Macaque-tropic HIV-1 derivatives provide a way to test whether antagonizing the activity of SAMHD1 is necessary for replication in OWM species.

## Conflict of Interest Statement

The authors declare that the research was conducted in the absence of any commercial or financial relationships that could be construed as a potential conflict of interest.
